# A tension offloading patch mitigates dermal fibrosis induced by pro-fibrotic skin injections

**DOI:** 10.21203/rs.3.rs-3915097/v1

**Published:** 2024-03-01

**Authors:** Heather E. Talbott, Michelle F. Griffin, Shamik Mascharak, Jennifer B.L. Parker, Maxwell M. Kuhnert, Jason L. Guo, Nestor M. Diaz Deleon, Christopher Lavin, Darren Abbas, Nicholas Guardino, Annah Morgan, Caleb Valencia, Asha Cotterell, Michael T. Longaker, Derrick C. Wan

**Affiliations:** 1 Hagey Laboratory for Pediatric Regenerative Medicine, Division of Plastic and Reconstructive Surgery, Department of Surgery, Stanford University School of Medicine; Stanford, California, 94305, USA.; 2 Institute for Stem Cell Biology and Regenerative Medicine, Stanford University School of Medicine, Stanford, California, 94305, USA.

**Keywords:** Fibrosis, bleomycin, diabetes, tension offloading, injection, insulin

## Abstract

Skin fibrosis is a clinical problem with devastating impacts but limited treatment options. In the setting of diabetes, insulin administration often causes local dermal fibrosis, leading to a range of clinical sequelae including impeded insulin absorption. Mechanical forces are important drivers of fibrosis and, clinically, physical tension offloading at the skin level using an elastomeric patch significantly reduces wound scarring. However, it is not known whether tension offloading could similarly prevent skin fibrosis in the setting of pro-fibrotic injections. Here, we develop a porcine model using repeated local injections of bleomycin to recapitulate key features of insulin-induced skin fibrosis. Using histologic, tissue ultrastructural, and biomechanical analyses, we show that application of a tension-offloading patch both prevents and rescues existing skin fibrosis from bleomycin injections. By applying single-cell transcriptomic analysis, we find that the fibrotic response to bleomycin involves shifts in myeloid cell dynamics from favoring putatively pro-regenerative to pro-fibrotic myeloid subtypes; in a mechanomodulatory *in vitro* platform, we show that these shifts are mechanically driven and reversed by exogenous IL4. Finally, using a human foreskin xenograft model, we show that IL4 treatment mitigates bleomycin-induced dermal fibrosis. Overall, this study highlights that skin tension offloading, using an FDA cleared, commercially available patch, could have significant potential clinical benefit for the millions of patients dependent on insulin.

## INTRODUCTION

Skin fibrosis is the accumulation of dense, non-functional, collagenous tissue in the dermis leading to loss of both normal form and function.(1) Fibrosis can occur in response to a variety of acute or chronic insults but is particularly common in the setting of insulin-controlled diabetes. Repeated insulin injection or pump delivery serves as a pro-inflammatory insult which, over time, leads to local skin changes including significant dermal fibrosis.(2, 3) The precise prevalence of injection site fibrosis is not known, particularly as fibrosis often accompanies and may be conflated with other local injection site skin changes such as fatty tissue changes. However, the prevalence of fibrosis is undoubtedly high. For instance, lipohypertrophy (LH), which typically has a fibrotic component,(4) is likely present in over half of all insulin users,(5) a staggering statistic in light of the fact that roughly 10 million Americans use insulin.(2) Fibrosis may also be increasing in prevalence with increased use of insulin pumps,(6) as both current and prior pump sites are associated with significantly increased local skin fibrosis.(3)

Injection site fibrosis is of particular concern not only because it is associated with visible/palpable skin changes, reduced patient satisfaction, and increased depression risk, but also because it can significantly influence insulin absorption from the fibrotic site.(2) We previously estimated that unnecessary insulin waste due to such impaired absorption likely costs the US healthcare system over $1 billion per year.(2) In addition, injection-/pump-induced skin changes make insulin uptake more variable and unpredictable, which impairs normal glycemic control and leads to increased risk of hypo-/hyperglycemia and even diabetic ketoacidosis, yielding further costs to individuals and healthcare systems.(7–10) However, despite the huge and growing prevalence of diabetes and insulin use, insulin injection-associated fibrosis remains an underappreciated problem. Currently, no adjunct therapies exist to prevent or reduce the fibrotic burden of insulin administration. A treatment or device capable of preventing injection-associated dermal fibrosis could improve clinical outcomes for the millions of insulin-reliant patients, and ultimately yield substantial cost savings for both these individuals and healthcare systems.

The role of mechanical forces and cell mechanical signaling in fibrosis, particularly wound healing/scarring, is well established.(11–18) The connection between wound fibrosis and mechanical tension can be intuitively appreciated: wounds closed under higher tension form more severe scars;(12) conversely, surgeons have long known to make incisions parallel to Langer’s lines of physiological tension along the skin (thus minimizing intrinsic tension across the wound).(19) The embrace device (Neodyne Biosciences, Inc.) was developed to therapeutically leverage this phenomenon to minimize scarring of incisional wounds. The device is a silicone patch that is pre-stretched, then shrinks back to its original size after being placed on the skin, thus applying a compressive force across the skin (perpendicular to the line of the wound) and reducing tension across the wound (Supplementary Fig. 1). This tension-reducing patch has been shown in multiple randomized clinical trials to significantly reduce scarring following surgery or scar revision.(20, 21) However, it is not yet known whether therapeutic approaches that physically reduce mechanical tension (e.g., tension-offloading patch application) could also reduce dermal fibrosis in other settings, such as following injection of a pro-fibrotic substance. While the precise nature of injury differs between these contexts, activated mechanotransduction has been shown to be a widely conserved mechanism of fibrosis across diverse organs, pathologies, and species,(22, 23) and prior studies have shown that distinct acute and chronic dermal fibroses may share common cellular and molecular etiology.(24, 25) Thus, we hypothesize that targeting tissue mechanics using a tension-offloading approach could also provide benefit for injection-induced dermal fibrosis.

Given the difficulties of studying insulin-induced fibrosis directly in animals, we modeled insulin’s local fibrotic effects with multiple intradermal injections of bleomycin, a small molecule known for its profound fibrotic effects. We performed our study in Red Duroc pigs, the gold standard animal model for studying dermal fibrosis as they exhibit a fibrotic/scarring injury response similar to that in humans.(26–28) We found that, histologically, tissue ultrastructurally, and biomechanically, application of a tension-offloading patch significantly prevented and reversed skin fibrosis caused by bleomycin injection. Single-cell transcriptomic analysis revealed that bleomycin injections were associated with a shift from a putatively pro-regenerative to a pro-fibrotic myeloid cell profile which was blunted by tension offloading. *In vitro* analysis supported that this pro-fibrotic myeloid shift was driven by mechanical tension and reversed by interleukin 4 (IL4). Finally, in a human foreskin xenograft model, IL4 blunted the fibrotic response to bleomycin, partially recapitulating the effects of tension offloading in our porcine model and collectively supporting a myeloid-mediated mechanism by which mechanical tension offloading may mitigate bleomycin-induced dermal fibrosis. Ultimately, aided by the relative safety and low risk of tension-offloading patch treatment (a therapeutic device that is already FDA cleared and commercially available), we believe this approach could have rapid clinical benefit to reduce the burden of insulin injection-induced skin fibrosis.

## RESULTS

### Developing a bleomycin-induced chronic fibrosis model to determine the effects of tension offloading on injection-induced fibrosis in red Duroc pigs

In order to fully appreciate the effects of tension offloading at the skin level on the development of dermal fibrosis following injection of a pro-fibrotic substance, we developed an experimental skin fibrosis model in red Duroc pigs. These pigs, due to their strong recapitulation of key features of human dermal fibrosis and scarring,(26–28) are considered the gold standard animal model for studying skin fibrosis and wound healing. Given the strong translational relevance of the process we are studying (dermal fibrosis following injection of a pro-fibrotic substance), as well as the fact that the treatment device we are using is already FDA cleared and optimal for rapid clinical translation, we wanted to ensure the conclusions of this study were as translationally relevant as possible. We used bleomycin to induce skin fibrosis as it has strong pro-fibrotic effects and, when injected locally in the skin, causes measurable fibrosis to develop in a predictable fashion without systemic adverse effects.(29, 30) Importantly, the skin changes resulting from bleomycin injection phenocopy key features of insulin injection-/pump-induced skin changes, including fibrosis with increased collagen content and inflammation.(3, 30, 31)

**Fig. 1:**
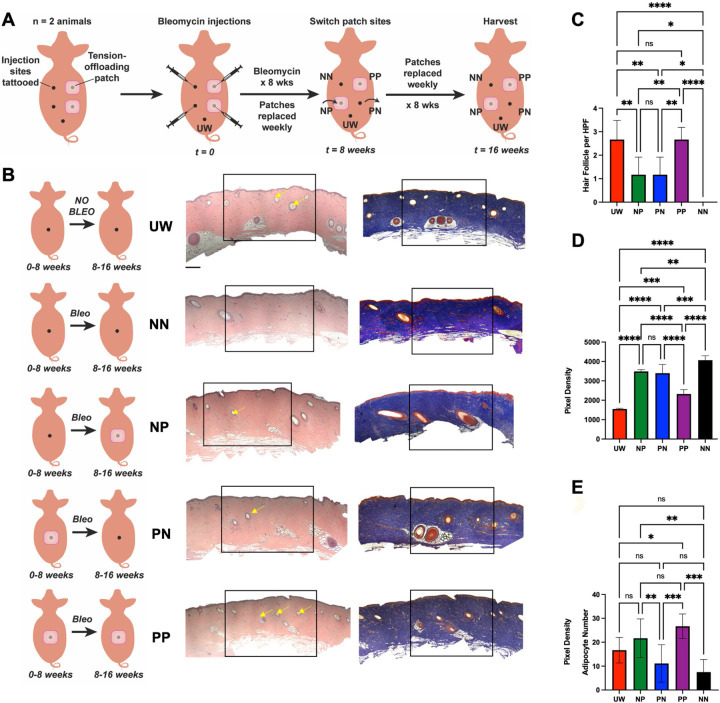
Histologic analysis shows that tension offloading at the skin level both prevents and reduces existing fibrosis induced by repeated bleomycin injections in red Duroc pigs. **(A)** Schematic depicting different treatment conditions in the red Duroc pig injection-induced fibrosis model. For the 16 week study period, each site received repeated intradermal bleomycin (bleo) injections over the initial 8 weeks and was subjected to one of four treatment regimens with tension-offloading patches: no patch for all 16 weeks (NN); patch for all 16 weeks (PP); no patch for the initial 8 weeks (during bleo injections), then patch for the latter 8 weeks (NP); or patch for the initial 8 weeks (during bleo injections), then no patch for the latter 8 weeks (PN). Healthy, unwounded (non-bleo-injected) skin sites (UW) were also harvested as a non-fibrotic control. **(B)** Schematic summaries of each experimental condition (left column), and representative hematoxylin and eosin (H&E; middle column) and Masson’s trichrome (MT; right column) histology from porcine skin in each experimental group. Yellow arrows, hair follicles. Black boxes indicate central injected regions based on which per high-powered field (HPF) quantification in (C-E) was performed. Scale bars, 250 μm. **(C-D)** Quantification of number of hair follicles (C) or collagen staining density (D) within HPF from MT images. (**E**) Quantification of adipocytes per HPF from H&E images. (B-E) *n* = 3 skin sites from each of 2 pigs were analyzed per group unless otherwise specified. (C-E) Data shown as mean ± standard deviation (SD). ns, not significant; *P < 0.05; **P < 0.01; ***P < 0.001; ****P < 0.0001.

Two individual pigs were used for this study. First, injection sites were designated on pigs’ dorsa by tattooing to ensure consistent location of bleomycin delivery. Experimental sites received intradermal bleomycin injections over the initial 8 weeks of the experimental period, then were maintained for the following 8 weeks and harvested after a total of 16 weeks (see Methods section for complete details). Each bleomycin-injected site was randomized to one of four conditions based on whether they were treated with a tension-offloading patch (“P”) or no patch (“N”) for each of the two halves of the study (conditions summarized in Fig. 1A):

1. Group “NN” had no patch for the entire 16 weeks of the experiment. This condition was a fibrotic control, as it was treated with bleomycin and did not receive any interventions that could potentially reduce fibrosis.
2. Group “PP” was treated with a patch for the entire 16 weeks of the experiment. This condition was intended to determine the maximum possible benefit of the tension-offloading patch with regards to mitigating bleomycin-induced fibrosis.
3. Group “NP” had no patch for the first 8 weeks, then was treated with a patch for the latter 8 weeks. This condition was intended to determine whether the patch could reverse or improve existing fibrosis that had developed in the initial 8 weeks of bleomycin application.
4. Group “PN” had a patch for the first 8 weeks, then no patch for the latter 8 weeks. This condition was meant to identify whether there was an erosion of benefit if the patch was removed for the latter 8 weeks.

In addition to these bleomycin-injected conditions, we also designated sites that did not receive any bleomycin or patch treatment and served as healthy, unwounded controls (“UW”). For patch-treated sites, tension-offloading patches were replaced weekly (reflecting recommended protocols for human use). Injections were done through the center of the patch (which did not disturb the attachment or placement of the patch). After a total of 16 weeks, pigs were euthanized, and all skin sites harvested. A necropsy report prepared by a blinded, trained veterinary pathologist at the Stanford University Veterinary Service Center (VSC) who was not involved in the study reported no grossly or histologically apparent lesions in any organ examined (lung, bone marrow, liver, spleen, kidney, gastrointestinal tract, heart, brain, skin [outside of experimental sites], or eyes), confirming that no systemic morbidity occurred with local bleomycin injections. Both longitudinal analyses over the course of the 16-week experimental period (e.g., mechanical testing of tissue elasticity using cutometer) and endpoint analyses at the time of harvest (e.g., histology and single-cell transcriptomic analysis) were performed in order to understand both the end result of different interventions and how tissue fibrosis was dynamic over the course of the study.

### Histologic analysis demonstrates that use of a tension offloading patch mitigates dermal fibrosis

We first used histologic analysis to compare skin among different treatment conditions at the time of harvest (16 weeks). Compared to normal skin that had not received any bleomycin (UW), bleomycin-injected skin *not* treated with a tension-offloading patch (NN) was clearly fibrotic by 16 weeks, with a dense, linearly aligned collagen architecture and the conspicuous absence of normal skin appendages (e.g., hair follicles) throughout the fibrosed area (Fig. 1B, **first vs. second row**, and Fig. 1C). In striking contrast, bleomycin-injected skin that had been treated with a tension-offloading patch for the entire duration of the experimental period (PP) was visually comparable to healthy, non-fibrotic skin (UW), with normal-appearing (less dense, more randomly aligned) collagen and numerous hair follicles present (Fig. 1B, **fifth row**). Quantification across specimens confirmed that the number of hair follicles per high-powered field (HPF) did not significantly differ between UW skin versus PP sites (Fig. 1C). Sites treated with a tension-offloading patch for only part of the experimental period (PN or NP) appeared to have an intermediate degree of fibrosis, with some hair follicles (but fewer than in either UW skin or PP sites; Fig. 1C) and intermediate-density collagen (Fig. 1B, **third and fourth rows**). Quantification of collagen staining density based on Masson’s trichrome (MT) staining confirmed that collagen density was highest (consistent with being the most fibrotic) in NN samples; conversely, PP samples had the lowest collagen density of all bleomycin-injected conditions and were the closest to UW skin (Fig. 1D). Finally, we noted that adipocyte numbers were decreased with bleomycin treatment, consistent with known effects of bleomycin. (Interestingly, while the effects of insulin treatment on adipose tissue are admittedly complex, a recent study established fat necrosis as a prevalent feature at insulin pump sites.(3)) Adipocyte numbers of PP and NP sites were similar to or greater than those in UW skin, while PN and NN sites had significantly decreased adipocytes compared to patch-treated conditions (Fig. 1E).

### Tension offloading prevents and mitigates fibrotic shifts in ECM ultrastructure

While differences in fibrosis were visually apparent from basic histology stains (Fig. 1), we sought to confirm these distinct outcomes using more objective, robust, and detailed methods than visual assessment. We previously developed a machine learning-based image analysis algorithm which, based on extracellular matrix (ECM)-stained (e.g., picrosirius red) histologic images, quantifies 294 distinct features of ECM ultrastructure on both local (e.g., fiber length, solidity) and global (e.g., branching, porosity) scales.(14, 32) This tool allows for unbiased, quantitative assessment of the complex architecture of fibrotic tissue, and allows for specimens to be objectively compared on the basis of their ECM ultrastructure. We have previously validated this image analysis pipeline in multiple fibrotic conditions across species (including mouse, pig, and human)(14, 32–36) and have shown that the fibrotic ECM features measured by our algorithm are clinically relevant (strongly predictive of highly clinically significant differences in patient survival in pancreatic cancer(32)).

**Fig. 2:**
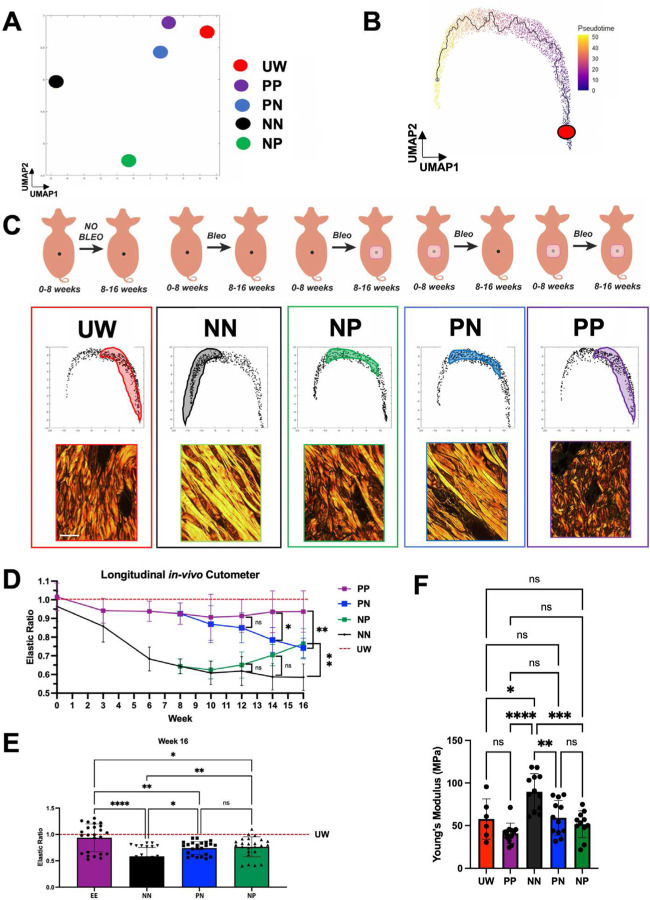
Tissue ultrastructure analysis and mechanical testing reveal that tension offloading prevents and rescues bleomycin injection-induced dermal fibrosis in red Duroc pigs. **(A)** Centroids of each experimental group’s extracellular matrix (ECM) ultrastructure parameters, mapped in uniform manifold approximation and projection (UMAP) space. **(B)** UMAP of ECM ultrastructure parameters corresponding to individual images across all experimental groups, with high pseudotime corresponding to increasingly disturbed/fibrotic ECM architecture. Red dot, trajectory root. **(C)** Top row, schematic summaries of each experimental condition. Middle row, UMAP recreated from (B), with shaded regions highlighting distribution of points corresponding to that experimental group across UMAP space. Bottom row, representative picrosirius red histology for each experimental group; scale bar, 10 μm. **(D-E)** *In vivo* cutometer measurements of skin elasticity for all experimental groups, normalized to measurements from UW skin regions, measured longitudinally over the course of the experiment (D) and at the 16 week experimental endpoint (E). **(F)** Young’s modulus values for skin samples from each experimental condition, calculated based on tensile strength testing of tissue harvested at 16 weeks. (A-F) *n* = 3 skin sites from each of 2 pigs were analyzed per group unless otherwise specified. (D-F) Data shown as mean ± standard deviation (SD). ns, not significant; *P < 0.05; **P < 0.01; ****P < 0.0001.

We applied this ECM ultrastructural analysis to picrosirius red-stained slides from each of our experimental specimens. We first performed uniform manifold approximation and projection (UMAP) based on parameters quantified from individual histologic images, then plotted the median/centroid for the subset of images corresponding to each condition. This analysis revealed that, overall, PP specimens had the most similar ECM to UW skin, suggesting they were the least fibrotic, whereas NN specimens were the most distinct from UW skin in terms of their ECM ultrastructure (Fig. 2A). We next calculated a pseudotime trajectory to enable individual images to be plotted along a spectrum from least fibrotic (i.e., most UW-like; low pseudotime value) to most fibrotic (most NN-like; high pseudotime value) and to facilitate comparison of overall ECM ultrastructure among experimental conditions (Fig. 2B). We then examined the distribution of images corresponding to each experimental condition along this pseudotime trajectory (Fig. 2C and Supplementary Fig. 2A). We confirmed that PP specimens most closely overlapped with UW specimens (low pseudotime/less fibrotic; Fig. 2C, **first vs. fifth columns**), indicating that patch treatment over 16 weeks maintained an ECM architecture similar to that of healthy, unwounded skin. This was supported by the close visual resemblance of representative picrosirius red images from these conditions, which showed thinner ECM fibers oriented in an interwoven, “basketweave”-like structure in both UW and PP skin (Fig. 2C, **first and fifth columns, bottom row**). UW and PP specimens were also quantitatively confirmed by our algorithm to have the greatest number of distinct fibers and fiber branchpoints (Supplementary Fig. 2B). In contrast, NN specimens minimally overlapped with UW, with NN skin exhibiting ECM parameters corresponding to high pseudotime values (indicating they were more fibrotic; Supplementary Fig. 2A**, top left panel**). Visually, picrosirius staining revealed dense, linearly aligned ECM fibers in NN skin (Fig. 2C, **first vs. second columns**) which lacked UW skin’s typical branching, interwoven/basketweave structure, supported by these specimens having significantly fewer distinct fibers and fiber branchpoints (Supplementary Fig. 2B). Collectively, this ECM analysis indicated that a distinct, fibrotic ECM architecture developed from bleomycin exposure in the absence of tension offloading, but that the tension-offloading patch restored ECM to a normal, UW-like architecture.

Sites treated with tension-offloading patches only for the first 8 weeks of the experimental period (PN) had ECM architecture that was quantitatively and visually intermediate between healthy skin (UW) and fully fibrotic skin (NN) (Fig. 2C, **fourth column** and Supplementary Fig. 2), consistent with a moderately fibrotic ECM architecture. This finding suggested that tension offloading for only 8 weeks was partially but not fully sufficient to prevent bleomycin-induced fibrosis. Interestingly, our ECM analysis also confirmed that NP sites, which were treated with the patch only during the latter 8 weeks of the experimental period (i.e., after the conclusion of bleomycin exposure), also had an intermediate degree of fibrosis (Fig. 2C, **third column**), suggesting that tension offloading may be at least partially capable of resolving existing/established fibrosis. This finding has important potential clinical implications, as the number of patients with existing skin fibrosis (e.g., from prior insulin injection sites) is, by nature, even greater than the already-large number of patients with newly developing fibrosis.

### Tension offloading prevents bleomycin-induced skin stiffening and restores healthy skin tissue mechanical properties

We next sought to interrogate the mechanical properties of skin in each experimental condition. Whereas normal skin is both strong and elastic, stiffening is a hallmark of fibrosis and can lead to discomfort, altered tissue appearance, and limited mobility.(37) Given our ECM ultrastructural findings (Fig. 2A–C and Supplementary Fig. 2) and the fact that tissue mechanics are closely related to ECM architecture (skin’s basketweave ECM structure lends it significant elasticity and pliability while maintaining strength), we hypothesized that bleomycin-treated sites in the absence of tension offloading would undergo gradual stiffening, whereas tension offloading would mitigate this stiffening and allow for maintenance or restoration of skin elasticity. To measure skin mechanical properties, we performed both longitudinal analysis over the 16-week experimental period (using a cutometer, which measures skin elasticity *in situ*) and endpoint analysis (both with cutometer and by subjecting harvested skin samples to Instron Instron mechanical strength testing, which calculates Young’s modulus, a measure of a material’s resistance to elastic deformation).

Cutometer analysis showed that over time, PP samples maintained elastic properties resembling those of UW skin’s, whereas NN samples became gradually stiffer from weeks 0–8 (during bleomycin administration), remained stiff from weeks 8–16, and were ultimately significantly stiffer than UW skin or PP samples at 16 weeks (Fig. 2D and Fig. 2E, purple vs. black), consistent with fibrosis established during the initial period of bleomycin injection. Interestingly, while NP samples, as expected, exhibited stiffening comparable to that of NN samples over the first 8 weeks, they diverged from NN samples and demonstrated increasing elasticity over the latter 8 weeks of the experiment (i.e., while they were being treated with the tension-offloading patch) and were ultimately significantly more elastic than completely non-patch-treated (NN) samples by 16 weeks (Fig. 2D and Fig. 2E, green), further supporting that the patch was capable of resolving existing fibrosis. PN samples remained relatively elastic (similar to either UW skin or PP sites) during the initial 8 weeks of the study, but then lost elasticity and became stiffer during the latter 8 weeks (after tension-offloading patches were removed) and were significantly less elastic than UW skin or PP sites by 16 weeks (Fig. 2D and Fig. 2E, blue), suggesting that continued patch treatment past the initial period of fibrotic insult may be required to fully prevent fibrotic changes. These cutometer results were all supported by mechanical strength testing of harvested tissue, which showed a significantly higher Young’s modulus (i.e., increased stiffness) in NN skin compared to UW or PP skin, and intermediate Young’s modulus for PN and NP sites at 16 weeks (Fig. 2F). Collectively, our tissue mechanical testing demonstrated that repeated bleomycin injections led to skin stiffening over time that was fully mitigated by tension offloading over 16 weeks, and that existing bleomycin-induced skin stiffening could be improved by ensuing application of a tension-offloading patch.

### Differential representation of myeloid cell subpopulations in the fibrotic versus non-fibrotic response to bleomycin injections

**Fig. 3:**
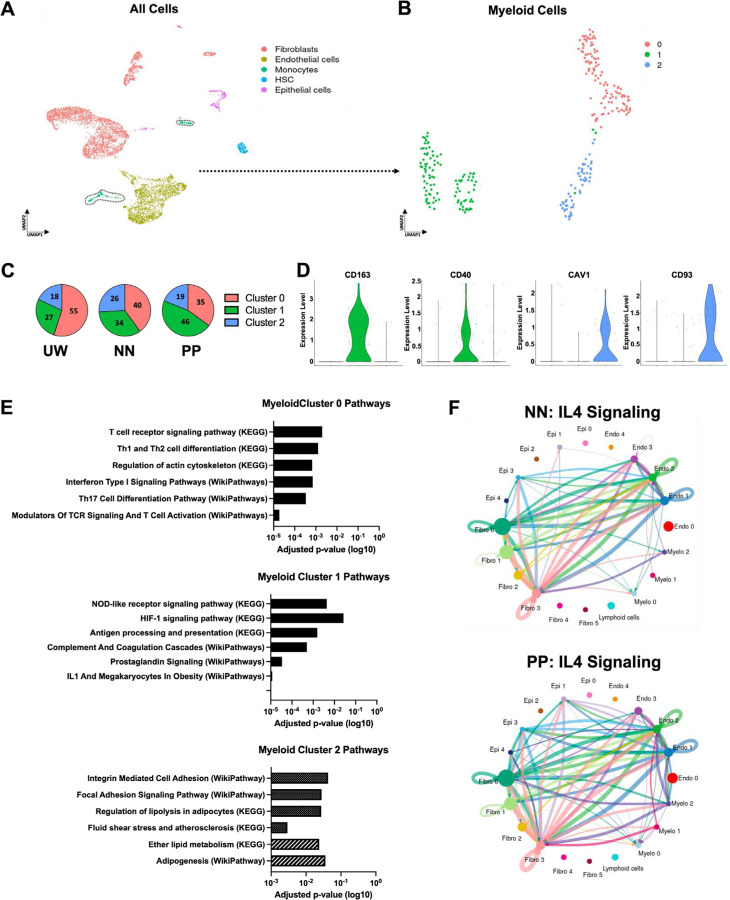
Single-cell transcriptomic analysis reveals that tension offloading alters myeloid cell dynamics in bleomycin injection-induced dermal fibrosis in red Duroc pigs. **(A)** UMAP of single-cell RNA-sequencing (scRNA-seq) data from porcine dermal cells across all experimental conditions and UW skin, colored by cell type (assigned *in silico*). Black dotted outlined region highlights cells classified *in silico* as myeloid cells which were used for downstream myeloid subcluster analysis. **(B)** UMAP of myeloid cells colored by Seurat cluster (0–2). **(C)** Relative representation (percentages) of myeloid cells in each indicated experimental condition (UW, NN, or PP skin) that belonged to Seurat clusters 0–2. **(D)** Violin plots depicting expression levels of cluster of differentiation (*CD*) 163, *CD40*, caveolin 1 (*CAV1*), and *CD93* for myeloid cells belonging to each Seurat cluster (0–2). **(E)** EnrichR analysis results for Pathways characteristic to cells in each myeloid Seurat cluster (0–2). **(F)** Inferred IL4 intercellular signaling network across cell types from NN (top) or PP (bottom) skin. Cells from *n* = 3 skin sites from each of 2 pigs were used for scRNA-seq analysis.

Highly encouraged by our histologic and tissue mechanical analyses showing that tension offloading patch treatment could effectively mitigate bleomycin-induced fibrosis, we next sought to further explore the cellular and molecular mechanisms behind these findings. To do so, we performed single-cell RNA-sequencing (scRNA-seq) on cells isolated from harvested UW skin, fully non-patch-treated/fibrotic (NN) skin, and fully patch-treated (PP) skin. Cell types were identified using computational tools to evaluate differentially expressed genes and associated functional pathways. Upon analyzing all cells sequenced, we found that our dataset included multiple cell types known to play important roles in fibrosis and regeneration, including fibroblasts, endothelial cells, monocytes, and epithelial cells (Fig. 3A), similar to those previously found in the setting of wound repair.(15) Each of these cell types was also found to comprise multiple transcriptionally-distinct subpopulations (Supplementary Fig. 3A). Our analysis allowed us to separately consider and analyze cells that were derived from unwounded, fibrotic control, or patch-treated skin sites (Supplementary Fig. 3B). Toward the goal of understanding how tension offloading affected cellular dynamics of fibrosis, we examined how the distribution of transcriptomically-defined cell populations differed between these treatment conditions. We first examined fibroblasts, as these are classically known to be the end mediators of fibrosis and our prior studies have implicated alterations in fibroblast subpopulation dynamics in influencing fibrotic versus regenerative skin healing outcomes.(14–16) Our scRNA-seq analysis identified six distinct fibroblast subclusters (Supplementary Fig. 3C). However, the proportions of these fibroblast subpopulations did not substantially differ between experimental conditions (Supplementary Fig. 3D), so we turned to other cell types in our dataset.

Intriguingly, when we focused our analysis on myeloid cells (monocytes) from our scRNA-seq dataset, we identified three transcriptionally distinct myeloid subclusters whose relative prevalence differed between the experimental conditions (Fig. 3B–C). Myeloid cluster 0 was significantly higher in UW skin than in either bleomycin-treated condition (NN or PP) (Fig. 3C, red region), suggesting this cluster may be specifically characteristic of uninjured skin. Clusters 1 and 2, in contrast, were more enriched in bleomycin-treated conditions (NN and PP) relative to UW skin (Fig. 3C, blue and green regions), indicating that these clusters may be more relevant to the injury/fibrosis response. Interestingly, myeloid cluster 1 was relatively enriched in PP compared to NN skin, while cluster 2 was enriched in NN relative to PP skin, suggesting that clusters 1 and 2 could be characteristic of a regenerative versus a fibrotic response to bleomycin injections, respectively.

### Defining molecular profiles of putative pro-fibrotic versus pro-regenerative myeloid cell subpopulations

In order to further characterize these myeloid subclusters of interest, we identified key genes whose expression was specific to each cluster. We found that cluster of differentiation (*CD*) 163 and *CD40* were highly expressed by cluster 1 but minimally expressed by all other myeloid subclusters, while caveolin 1 (*CAV1*) and *CD93* specifically marked cluster 2 myeloid cells (Fig. 3D). This finding was important as it suggested that these genes and their corresponding proteins could potentially be leveraged to enable prospective isolation and/or specific manipulation of each myeloid cell subpopulation. In order to verify that differential representation of the myeloid cell subclusters in our scRNA-seq dataset was also reflected by differential representation of these cell subsets between experimental conditions *in vivo*, we performed immunofluorescent (IF) staining for CD163, CD40, CAV1, and CD93 in histologic sections of UW, PP, and NN skin, along with co-staining for CD45 to specifically stain immune cells (Supplementary Fig. 4A). (As our scRNA-seq analysis indicated that myeloid cells/monocytes were the predominant/exclusive immune population present in skin samples at week 16 [Fig. 3A], CD45 should serve as a surrogate marker for myeloid cells in these samples.) Consistent with myeloid cluster 1’s relative enrichment in PP samples by scRNA-seq, CD45^+^CD163^+^ and CD45^+^CD40^+^ cells were significantly upregulated in PP skin, while CD45^+^CAV1^+^ and CD45^+^CD93^+^ cells were increased in fibrotic NN skin, consistent with myeloid cluster 2 being upregulated among cells from NN samples on scRNA-seq (Supplementary Fig. 4B).

We next sought to more broadly characterize gene expression programs characteristic of each of these myeloid cell clusters. To do so, we performed Gene Ontology (GO) pathway enrichment analysis for cells from each cluster (Fig. 3E). Cluster 1 (enriched in PP skin) highly expressed genes related to known inflammatory and immune cell pathways as well as angiogenesis, including the GO terms “HIF-1 signaling pathway,” “Antigen processing and presentation,” and “Complement and coagulation cascades” (Fig. 3E, **second panel**). In contrast, cluster 2 (higher in NN skin) was enriched for GO terms related to mechanotransduction signaling, including “Integrin mediated cell adhesion” and “Focal adhesion signaling pathway,” as well as terms related to adipogenesis and lipid signaling, such as “Regulation of lipolysis in adipocytes,” “Ether lipid metabolism,” and “Adipogenesis” (Fig. 3E, **third panel**).

In order to further interrogate intercellular signaling and how cell-cell crosstalk involving these myeloid cell clusters may differ under distinct experimental conditions, we applied the computational tool CellChat, which infers intercellular communication patterns based on scRNA-seq data(38) (Supplementary Fig. 5A). CellChat revealed intercellular communication axes/signaling pathways that were differentially active in PP versus NN skin; for instance, cell crosstalk involving interleukin (*IL*) 16, tumor necrosis factor (*TNF*), and *NOTCH* was relatively increased in NN fibrotic skin, whereas hepatocyte growth factor (*HGF*), growth differentiation factor (*GDF*), and semaphorin 7 (*SEMA7*) crosstalk was increased in PP skin (Supplementary Fig. 5B). Notably, *IL4*-related cell-cell signaling was one of the pathways most significantly enriched in the PP condition (Supplementary Fig. 5B). Specifically, *IL4* intercellular signaling involving myeloid cell subclusters (including myeloid-fibroblast crosstalk) as well as epithelial cells (especially between epithelial cells and fibroblasts) was upregulated in PP compared to NN skin; in particular, cell crosstalk between myeloid cluster 1 and fibroblasts was absent in NN skin but highly enriched in PP skin (Fig. 3F).

### Recombinant IL4 prevents a mechanical tension-induced pro-fibrotic myeloid shift *in vitro*

Given the pattern of *IL4* upregulation in PP compared to NN skin, as well as the known important role of IL4 signaling in tissue repair,(39) we sought to explore whether IL4 itself was sufficient to influence porcine myeloid cell phenotype. We applied a mechanomodulatory hydrogel system to robustly investigate the relationship between IL4, cell mechanical environment, and myeloid cell phenotype *in vitro* (Supplementary Fig. 6A). In this platform, cells of interest (in this case, fluorescence-activated cell sorting [FACS]-isolated porcine CD45^+^ cells) are encapsulated and cultured in a hydrogel, which can then undergo stretching to apply controlled strain to the contained cells. Upon comparing cells cultured under stretched versus unstretched conditions, we found that while unstretched control cells expressed abundant CD163 (a marker of the putative pro-regenerative/anti-fibrotic myeloid cluster 1 phenotype), applying mechanical strain effectively abrogated the cells’ CD163 expression (Supplementary Fig. 6B–C, “Control Stretch” vs. “Control Unstretched). This *in vitro* finding suggested that mechanical strain alone was potentially sufficient to drive a shift from a relatively non-fibrotic to a more-fibrotic myeloid phenotype, whereas the putatively pro-regenerative/non-fibrotic myeloid phenotype dominated in the absence of mechanical tension. This in turn supported the concept that mechanically-driven differences in myeloid subpopulation bias could contribute to the observed fibrotic versus non-fibrotic outcomes of bleomycin injections under conditions of physiologic versus reduced mechanical tension *in vivo*.

We next sought to directly determine whether IL4 could influence this mechanically driven, pro-fibrotic myeloid shift *in vitro*. To do so, we exposed cells to recombinant IL4 protein and cultured either with or without stretch. Notably, application of IL4 significantly increased the presence of CD163^+^ cells in stretched gels compared to control (non-IL4-treated) stretched gels (Supplementary Fig. 6B–C, “IL-4 Stretch” vs. “Control-Stretched”), indicating that IL4 was able to at least partially prevent the switch to a more-fibrotic myeloid cell phenotype typically observed in response to mechanical strain.

### Recombinant IL4 reduces bleomycin-induced fibrosis in a human skin xenograft model

**Fig. 4:**
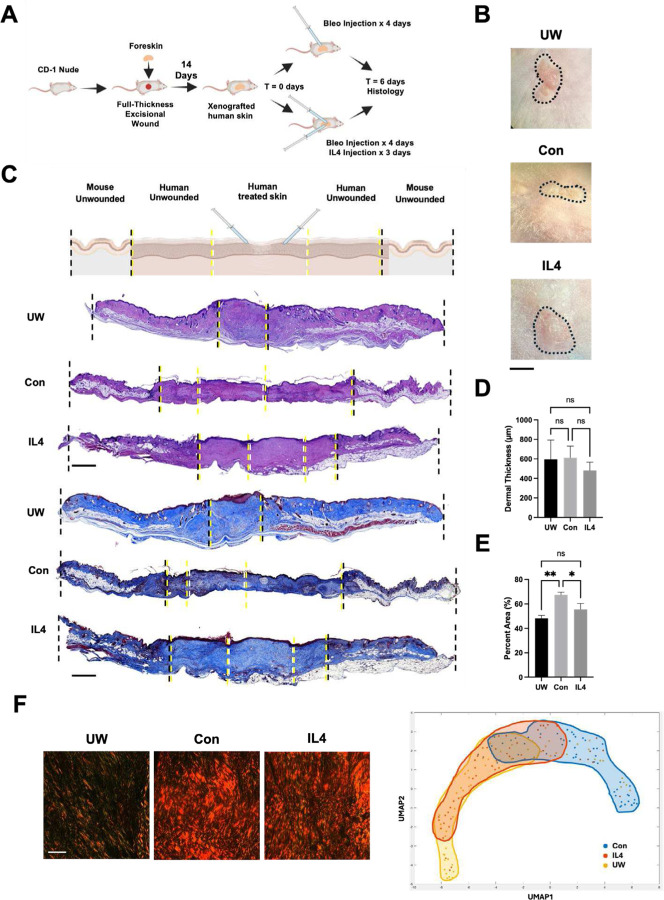
IL4 reduces bleomycin injection-induced fibrosis in a human skin xenograft model. **(A)** Schematic of human foreskin xenografting and bleomycin-induced fibrosis experiments. **(B)** Gross photographs of unwounded/non-bleomycin-injected (UW), bleomycin-injected (fibrotic control; Con), and IL4-treated, bleomycin-injected (IL4) xenograft skin at the time of harvest (6 days following initial bleo injections). Black dotted outlined region indicates boundary/edge of xenograft versus surrounding mouse skin. **(C)** Top row, schematic depiction of different regions of skin shown in xenograft histology below. Bottom rows, representative H&E (rows 2–4) and MT (rows 5–7) histology of xenograft specimens corresponding to each experimental condition, with dotted lines representing boundaries of regions labeled on schematic (first row). **(D)** Quantification of xenograft dermal thickness from H&E histology. **(E)** Quantification of xenograft collagen staining intensity from MT histology. **(F)** First three panels, representative picrosirius red histology from each xenograft experimental condition. Right panel, UMAP of quantified ECM ultrastructure parameters for xenograft specimens, with shaded regions highlighting experimental group trends. (B-F) *n* = 3 xenografts analyzed per group. (D,E) Data shown as mean ± standard deviation (SD). ns, not significant; *P < 0.05; **P < 0.01. Scale bars, 500 μm (B), 25 μm (F).

Given our promising *in vitro* results and scRNA-seq findings suggesting that IL4 could block myeloid cells from transitioning from a pro-regenerative to a pro-fibrotic phenotype, we sought to determine whether IL4 could similarly prevent fibrosis *in vivo*. To address this question, we employed a human foreskin xenograft model, which we have previously validated and used to study the effects of potential anti-fibrotic interventions.(36, 40) We engrafted human foreskin samples onto the dorsum of immune-deficient mice (CD-1 nude – to prevent graft rejection), as previously reported,(36) then subjected xenografts to repeated injections of bleomycin with or without adjunct injections of recombinant IL4 protein; other grafts were made and not injected with bleomycin, as an UW/non-fibrotic control (Fig. 4A–B). Grafts were then harvested for histologic and quantitative ECM analysis.

Validating that our model yielded fibrotic changes in xenograft skin, H&E histology of grafts showed that, overall, the central region of the xenografts had more compact/dense, fibrotic-appearing tissue architecture in control bleomycin-injected (“Con”) compared to healthy UW xenografts (Fig. 4C, **second through fourth rows**, “UW” vs. “Con”). This impression was further supported by MT staining, which demonstrated substantially denser-appearing and more collagenous dermal tissue, with more densely packed and linearly-aligned collagen fibers, in bleomycin-injected compared to UW xenografts (Fig. 4C, **fifth through seventh rows**, “UW” vs. “Con”). Dermal thickness did not significantly differ between conditions at this timepoint (Fig. 4D), but quantification of MT staining confirmed that control bleomycin-treated xenografts were significantly more collagenous compared to UW xenografts (Fig. 4E, “UW” vs. “Con”).

Notably, these fibrotic changes were blunted by IL4 treatment. Bleomycin-injected xenografts that were treated with IL4 had less compact-appearing tissue architecture and appeared less dense and collagenous/fibrotic on histology compared to control bleomycin-injected xenografts (Fig. 4C, “Con” vs. “IL4”). IL4-treated specimens were also, quantitatively, significantly less collagenous than control bleomycin-injected specimens, instead exhibiting MT collagen staining density that did not significantly differ from that of unwounded skin (Fig. 4E). We also applied our quantitative ECM ultrastructure analysis pipeline to xenograft specimens. Visually, picrosirius red staining showed that IL4-treated specimens, while still more fibrotic-appearing than UW skin, appeared less fibrotic than control bleomycin-injected specimens, with thinner and less dense ECM fibers (Fig. 4F, first three panels). Quantitatively, control bleomycin-injected skin exhibited ECM parameters that minimally overlapped with those of UW skin, whereas IL4-treated specimens had ECM parameters that largely overlapped with those of UW skin (Fig. 4F, right panel), consistent with IL4 treatment restoring normal/non-fibrotic, UW-like dermal architecture.

## DISCUSSION

The role of mechanical tension in fibrosis has been extensively explored in the context of wound healing and scarring. While the contribution of mechanical forces to other fibroses is beginning to be understood – for instance, a mechanically activated, pro-fibrotic fibroblast subtype has also been shown to play a central, conserved role in the fibrotic reaction around multiple cancers(22) – whether this fact can be clinically leveraged, as it already has been in wounds with the development of tension-offloading dressings,(20, 21) remained to be seen.

Dermal fibrosis following chronic insulin administration is a major issue for insulin-dependent diabetic individuals, as it causes not only direct clinical issues (e.g., discomfort and cosmetic deformation) but also problems with insulin absorption and glycemic control. We have previously postulated that physical tension offloading at the skin level, such as through the use of an elastomeric adhesive patch, could potentially target multiple harmful sequelae of insulin administration in the skin, most importantly chronic fibrosis.(2) In the present study, we have now found experimentally that a tension-offloading patch (the embrace device, Neodyne Biosciences, Inc.) effectively mitigates fibrosis in the context of repeated pro-fibrotic (bleomycin) injections. We were easily able to perform repeated bleomycin injections directly through the patch, and we envision that insulin injections could be performed, or a pump cannula placed, through the patch as well.

Our experimental findings could have important clinical implications. The patch used in our study is already FDA cleared and commercially available, which should facilitate translation. The prevalence of insulin-induced skin fibrosis is likely underappreciated, as research has largely focused on more visible changes such as LH. Notably, though, these fatty changes frequently have a significant fibrotic component,(4) and clinical studies suggest that fibrosis is likely a more permanent complication of insulin administration compared to simple LH.(5) Based on these facts and recent clinical studies,(3) we would postulate that fibrosis is likely an issue in the majority of insulin pump users as well as individuals who require regular insulin injections.

The results of our study also have implications for the optimal clinical implementation of tension offloading for insulin users. The PP condition, wherein a tension offloading patch was applied both throughout the fibrotic induction period (during bleomycin injections) and in the following weeks, was most effective in completely preventing bleomycin injection-induced fibrosis. The limited effectiveness of the PN condition, wherein the patch was only used during the period of active bleomycin injections, supports that fibrosis can continue to develop even after the immediate fibrotic insult is removed. Combined, these results suggest that the most effective clinical treatment modality may be patch use at the injection/pump site both during and for a period after insulin administration. However, the NP condition was also partially effective, suggesting that patch use even on an existing fibrotic site (i.e., a former insulin injection/pump site) could help to reduce the resultant fibrotic burden. Ultimately, clinical trials will be instructive in determining the precise window around insulin treatment, if any, in which tension offloading is most effective for ultimately reducing the fibrotic burden.

In this study, we identify a possible mechanism, involving perturbations to myeloid cell subcluster representation, by which tension offloading could reduce fibrosis following repeated bleomycin injections. Specifically, we found that tension may drive a transition from a relatively non-fibrotic to a pro-fibrotic myeloid subtype, but this transition could be prevented by IL4, and that IL4 application *in vivo* (in a human xenograft model) could prevent bleomycin-induced skin fibrosis. Of note, these effects of IL4 treatment on fibrosis were only partial (i.e., did not fully recapitulate the effects of tension offloading). One possible reason is that the timescale of our xenograft IL4 experiments was significantly shorter than that of porcine tension-offloading experiments, and more complete resolution of fibrosis by IL4 could be seen on a longer timescale. However, another possibility is that the IL4-/myeloid-dependent mechanism is only one component of the fibrotic reaction that occurs in the skin following bleomycin injection, as fibrosis is known to be a complex and multifactorial process involving diverse cell types.(15, 41) Looking in more detail at pathways that CellChat analysis uncovered as being differentially enriched between the different experimental conditions, there were other cell crosstalk pathways enriched in NN skin that have been previously found to be involved in fibrosis, including *IL16*(42) and *NOTCH*.(43) The implication of adipocyte-related pathways in the putatively pro-fibrotic myeloid cluster (Fig. 3E) may also be of interest: fat and fibroblasts/fibrosis are increasingly understood to have an intimate relationship;(40, 44, 45) lipid dysregulation is common at insulin injection sites and often co-occurs with fibrosis;(4–6) and we have previously postulated that, given current knowledge about the relationship between mechanical forces and adipocytes, tension offloading could be beneficial for both fibrosis and lipodystrophy in the setting of insulin use.(2) Conversely, other cell crosstalk pathways enriched in PP skin have been implicated by other studies as involved in tissue repair and/or possibly anti-fibrotic, including *HGF*,(46, 47) occludin (*OCLN*),(48) and *GDF*.(49–51) One further limitation of our study is that we were only able to apply scRNA-seq analysis to porcine tissues at the time of harvest (16 weeks); other mechanisms could potentially be uncovered by future studies examining skin cells at multiple timepoints to understand how the fibrotic process, and its mitigation by tension offloading, are dynamic over time.

We elected to use bleomycin in our model because the complicated nature of studying diabetic animals, and the substantial physiological effects of insulin outside of the skin, pose a significant logistical challenge to directly modeling local skin fibrosis induced by insulin injection or pump use. While we found that repeated bleomycin injections recapitulated key known features of insulin-induced dermal fibrosis (e.g., collagen buildup, skin stiffening), it must be acknowledged that insulin-induced fibrosis could differ mechanistically from bleomycin-induced fibrosis, and thus our conclusions regarding the efficacy of tension offloading may or may not hold. (It is also possible that a tension offloading approach could be even *more* effective in the setting of insulin use, as bleomycin is likely more strongly fibrotic than insulin.) Given the evidence for a conserved role of tissue mechanics and mechanotransduction signaling across diverse fibroses, and strong evidence from the present study, we believe a tension-offloading approach would also be effective in the setting of insulin use; but, this question can only be directly experimentally addressed by further diabetic animal studies and/or human trials. Since the patch used in this study is already known and FDA cleared to be safe on wounds and is readily commercially available, we would argue that human clinical trials could be a reasonable next step toward this end. Beyond insulin-induced skin changes, this study could pave the way for a non-invasive, skin-level tension offloading approach to be applied in diverse other settings of skin fibrosis.

## METHODS

### Animal skin fibrosis models

#### Pigs:

Two red Duroc pigs (one female, one male; both 12 weeks old) were obtained from Pork Power Farms (Turlock, CA) and allowed one week to acclimatize to facilities prior to the start of experiments. All pigs were maintained at the Stanford University Research Animal Facility under an approved protocol (APLAC #33742) in accordance with the Stanford University Animal Care and Use Committee Guidelines.

#### Porcine bleomycin and patch treatment:

Pigs were placed under general anesthesia and skin sites were marked by intradermal injection of a small volume of India ink prior to the first bleomycin injection. Sites were spaced at least 6 inches apart to prevent crossover effects between sites. A total of 12 bleomycin-injected sites were studied per pig (24 bleomycin-injected sites total) in addition to 3 non-injected sites per pig (non-fibrotic/uninjured control). Sites were randomly assigned to experimental conditions (NN, NP, PN, PP, or UW) to avoid bias of results by anatomical confounding factors (e.g., medial/lateral, or rostral/caudal differences in propensity to fibrosis).

Bleomycin dosing was determined by scaling from mouse studies by relative weight.(30) Bleomycin (USP, Millipore Sigma) was resuspended in sterile saline (0.2 μg/μl) and 150 μg (750 μl) was injected intradermally per site at tattooed locations, three times a week for two weeks, then once weekly for the following six weeks (total of 12 injections per site). We confirmed, using India ink injections into separate (distinct from the experimental sites) skin regions, that the localization of the injections was in fact intradermal, and that intradermally injected material ultimately was largely retained intradermally but also extravasated to the subcutaneous layers (Supplementary Fig. 7). For sites receiving tension offloading treatment, tension offloading patches (Embrace Active Scar Defense, size Medium [2.4 inch], Neodyne Biosciences) were purchased (Amazon) and applied per manufacturer instructions, centered over the tattooed injection sites. For patched sites, injections were performed through the center of the patch.

A custom-fitted neoprene jacket (Lomir Biomedical) was worn by all experimental animals to prevent animals from removing the patches between changes. After a total of 16 weeks following the initial bleomycin injections, pigs were euthanized and all skin sites harvested with sharp surgical scissors.

#### Human foreskin xenograft fibrosis model:

Neonatal human foreskin samples were collected after circumcision at the Lucile Packard Children’s Hospital at Stanford under a Stanford University Institutional Review Board (IRB) approved protocol (IRB #45219; APLAC 11048). Foreskin was first prepared by removal of all subcutaneous tissue, including muscle, and divided into 1 cm sections to be grafted. Grafting of the foreskin onto nude mice was performed under anesthesia with 2–4% isoflurane (Product:502017, MWI Veterinary Supply Co.^®^; Boise, ID) at a rate of 2 L/min. While anesthetized, Betadine Surgical Scrub Veterinary (Avrio Health L.P.^™^, Stamford, CT) and sterile alcohol prep pads (FisherScientific^™^, Pittsburgh, PA) were used to prepare the dorsum. A 1.2 cm full-thickness excision of dorsal skin was created with forceps and sharp scissors. The foreskin graft was then secured into the full-thickness wound using 5–0 monofilament Nylon sutures (Medtronic, Minneapolis, MN). Tegaderm film (3M^™^, Saint Paul, MN) was used for postoperative dressings. Dressings were maintained for five days postoperatively before being removed.

At two weeks postoperatively, complete engraftment was established, and the nylon sutures were removed. Using an insulin syringe, 20 μL of bleomycin sulfate (Millipore Sigma, Cat: 1076308; 10 mg/mL in sterile phosphate buffered saline [PBS]) were injected intradermally in the center of the graft per day for four days. To ensure the injection site remained consistent across the treatment, 5 μL of 0.2% India ink solution (Fisher Scientific, Cat: 15433679) was injected intradermally 2 mm laterally and 2 mm longitudinally from the bleomycin-injected site. On the first day of injection, and for the two days following (total 3 days), half of the mice also received 20μL intradermal injection of recombinant human IL4 protein (R&D Systems, Cat: 204-IL; 20 ng/μL in PBS). Mice were harvested at day 6 following the first bleomycin injection for histological analysis.

Mice were housed at the Lokey Stem Cell Research Building Barrier Facility (SIM1 Barrier) at Stanford University per Stanford APLAC guidelines, under the supervision of the Veterinary Service Center (VSC).

### Skin histology

#### Tissue processing and basic histology:

Collected tissue was fixed in 4% paraformaldehyde (PFA) at 4 °C overnight. Samples were then washed with PBS and placed in an automated Tissue Processor (ThermoFisher Scientific^™^, Waltham, MA). Using a ThermoFisher Histostar Tissue Embedding station, processed tissue was imbedded into paraffin blocks. Paraffin blocks were then sectioned at 8μm thickness onto Superfrost/Plus adhesive slides (ThermoFisher Scientific^™^, Waltham, MA). Slides were then stained with hematoxylin and eosin (Cat: H-3502; Vector Laboratories, Burlingame, California), Masson’s Trichrome (ab150686; Abcam^®^, Waltham, MA), and Picrosirius Red (ab150681; Abcam^®^, Waltham, MA). Slides were imaged with a 40x objective using a MoticEAsyScan One slide scanner.

#### Collagen density quantification:

Masson’s Trichrome stained specimens were used for collagen density measurements. Integrated density measurements of stained collagen were measured via ImageJ’s color deconvolution plugin and blue pixel distribution was quantified using ImageJ’s Color Detect macro.

#### Dermal thickness quantification:

Hematoxylin and eosin-stained slides were used for dermal thickness quantification. Dermal thickness was determined by measuring the distance between the basal layer and the boundary of the hypodermis and measured using ImageJ.

### ECM ultrastructure quantitative analysis

Picrosirius red stained slides were imaged using polarization microscopy at 40x magnification. Each group (*n* = 3 samples per group) were imaged at random unique locations (300 locations for each pig group, 50 for each xenograft group), and then run through using an in-house image-processing algorithm as previously describe.(52) In brief, color deconvolution was performed to characterize collage fibers by absorbance in red, green and blue channels. Ortho-normal transformation followed to the contribution of each color to a given image. The red and green images produced represented mature and immature extracellular matrix (ECM) fibers, respectively, and were analyzed separately. Matlab-based script was applied to conduct downstream analysis, which included noise reduction, preferential selection for smooth regions with low variance, and “skeletonization” of images. This script also enables quantification of fiber length, width, persistence, alignment, and dimensionality.

Using quantified matrix values identified from the ultrastructure algorithm, all datapoints were run through the *DDRTree* algorithm, with a minimum spanning tree and minimum branch length of 30. Pseudotime values were then assigned to each ultrastructural datapoint based on their geodesic distance to the root point, representing baseline histological architecture. Centroids were calculated as the median value for all images from a given experimental group.

### Biomechanical testing

#### Cutometer:

The Cutometer^®^ Dual MPA 580 (Courage+Khazaka, Köhn, Germany) was used to measure biomechanical skin properties of porcine skin *in vivo* with Mode 1 (time-strain mode) on the MPA Q software with a Windows^®^ operating system. With the force of probe suction set at 300mbar (30kPa), and applying the 8mm aperture probe, measurements were taking in a humidity and temperature-controlled environment (temperature maintained at 23°C and humidity regulated at 35%). Animals were acclimatized to the regulated environment for at least one hour prior to testing. To remove any residual sweat, oil, hair, or any other debris on the skin surface that could interfere with cutometer reading, hair was removed from the animal at the testing sites and these sites were cleaned with alcohol preparation swabs and allowed to dry. For the unwounded (UW) skin group, normal skin between conditional sites was measured for this group in order to minimize the impact of anatomic difference in native skin properties. For experimental groups, measurements were taken in the center of each site.

#### Instron mechanical strength testing:

An MTS Bionix 200 (MTS Systems, Eden Prairie, MN) was used for *ex vivo* biomechanical testing. Strips of porcine skin from each experimental group were collected following harvest and loaded onto the MTS device with an Interface SM-10 force transducer. Samples received constant mechanical stress until they avulsed. Stress-strain curves generated with the software were then used to calculate Young’s moduli. All samples were subjected to initial testing, but measurements were discarded from any samples that slipped on the machine grips during measurement (leading to erroneous measurements) or exhibited an abnormally-shaped stress-strain curve (indicative, in our extensive past experience, of machine and/or sample loading error)

### Single-cell RNA-sequencing

#### Single cell isolation from porcine skin:

Sixteen weeks following bleomycin injection, in parallel to tissue collection for histology and mechanical testing, portions of the skin were saved for downstream single-cell RNA-sequencing (scRNA-seq). Using sharp surgical scissors, skin was mechanically digested and then enzymatically digested with Collagenase II (ThermoFisher, Cat: 17101015) and IV (ThermoFisher, Cat: 17104019) in DMEM-F12 (GIBCO^™^, Fisher Scientific, Hampton, NH). Samples were placed on an orbital shaker for 90 minutes at 150 RPM at 37°C. FACS buffer was added to quench the enzymatic digest, and samples then passed through a 70μm cell strainer to remove large debris. Cells were centrifuged at 1500 RPM at 4°C, resuspended in FACS buffer, passed through a 40μm strainer, and counted. Cell suspensions were submitted to the Stanford Functional Genomics Facility for library preparation (10x Chromium Single Cell platform; Single Cell 3’ v3, USA) and sequencing.

#### Data processing, fastq generation, and read mapping:

Cell Ranger (10X Genomics; version 3.1)’s mkfastq was used to convert base calls to reads and align data against Cell Ranger’s human reference genome using the count function with SC3Pv3 chemistry. Maximum percent mitochondrial RNA was capped at 15%. A maximum of 7,500 unique genes and 85,000 RNA counts were used for downstream analysis.

#### Data normalization and cell subpopulation identification:

Unique molecular identifiers (UMIs) from each barcoded cell were normalized using a scale factor of 10,000 UMIs/cell. Using the R package Seurat (version 4.0.5), these data were natural log transformed with a pseudocount of 1.(53) The first 15 principal components of normalized data were used for uniform manifold approximation and projection (UMAP) analysis.

#### CellChat receptor-ligand analysis:

The CellChat platform was applied to cell-cell interactions.(38) CellChat’s Shiny App for its Cell-Cell Communication Atlas Explorer was applied to our scRNA-seq Seurat object in R. Cells were binned based on SingleR-defined cell types, and default parameters used to identify Secreted Signaling, ECM-Receptor, and Cell-Cell Contact relationships.

### Immunohistochemistry

Immunohistochemistry (IHC) was conducted on 8 μm thickness sectioned paraffin slides. Following incubation for one hour at 50 °C, slides were washed twice in Tween 20 (Sigma-Aldrich^™^, St. Louis, MO) followed by one wash in PBS. Slides were then blocked for 1 hour with Power Block (Biogenex^™^, Fremont, CA) prior to addition of the following primary antibodies: CD45 (Abcam, Cat: ab8216), CD163 (Cat: PA5–78961), CD93 (Cat: PA5–52930), CAV1 (Cat: ab214448), CD40 (Cat: 2246639), COL1 (Cell Signaling, Cat: 72026S). Slides were then incubated for 1 hour with Alexa Fluor 488, 594, or 647-conjugated anti-rabbit, anti-rat, or anti-mouse antibodies (Invitrogen, Waltham, MA). Finally, slides were mounted in Fluoromount-G mounting solution with DAPI (ThermoFisher Scientific^™^, Waltham, MA). Images were acquired with a LSM880 inverted confocal, Airyscan, AiryscanFAST, GaAsP detector upright confocal microscope.

### *In vitro* mechanical stretching of porcine macrophages

#### Harvesting cells for fluorescence-activated cell sorting:

Following euthanasia, additional unwounded porcine skin tissue was dissected and washed once in PBS. Wounded tissue was minced using sharp surgical scissors. Minced skin tissue was then enzymatically digested using a 1:1 ratio of Collagenase Type IV (ThermoFisher, Cat:17104019) and Collagenase Type II (ThermoFisher, Cat:17101015) at a concentration of 1500U/ml in DMEM (ThermoFisher, Cat:10569010) for 90 minutes at 37 °C. Following enzymatic digestion, fluorescence-activated cell sorting (FACS) buffer was used as a quenching agent and wash buffer, and the resuspension then put through 70 μm cell strainers. Cells were pelleted at 1500 rpm for 5 minutes at 4 °C and then resuspended in 150uL of FACS buffer for primary antibody staining with CD45 (ThermoFisher, Cat:48045942). Following further washing, secondary antibody staining was performed. Following staining cells were washed again with FACS buffer, and DAPI (4′,6-diamidino-2-phenylindole) (BioLegend, Cat: 422801) was added to label dead cells. A BD II FACSAria machine was used for FACS sorting and analysis. In vitro *mechanical stretching of porcine macrophages:*

FACS-isolated porcine macrophages were used to populate the collagen I hydrogels as per a previously published protocol.(54) In brief, a solution of 2 mg/mL collagen I (Advanced Biomatrix, Cat:5005), 0.8x MEM (ThermoFisher, CAT:11430030) in 16 mM HEPES (Sigma, CAT:83264), and suspended porcine macrophages (concentration 50,000 cells/mL) in DMEM (ThermoFisher, Cat:10569010) were pipetted into cruciform-shaped PDMS (DOW, Cat:2646340) molds and allowed to gelate before removing the mold. After 24 hours, formed hydrogels were subjected to either 10% equibiaxial strain or no strain by movement of metal anchor pins in each of the four arms of the hydrogel. Strain quantification was confirmed by FIJI analysis of TiO_2_ dye marks on the central region of each hydrogel.

Media containing recombinant interleukin 4 (IL4) protein (IL4; 10 ng/μL; R&D Systems, Cat: BT-004) or vehicle control (PBS) was also added at the time of stretch. Hydrogels were kept for 48 hours days. Gels were fixed whole in 4% PFA (ChemCruz, Cat:281692) after being washed in PBS for immunohistochemical analysis.

#### Immunocytochemistry processing for gels:

Following fixation, gels were dissected into 2mm × 2mm pieces and placed into a 96 well plate. Gentle agitation was provided during staining via a platform shaker at 23°C. Gel pieces were washed in PBS for 30 minutes. They were then permeated with 0.1% TritonX-100 in PBS (ThermoFisher, Cat:85111) for 30 minutes. Gels were then blocked with PowerBlock (BioGenex, Cat: HK085-GP) for 1 hour. Following blocking, primary antibody was made up in a 1:1 solution of 0.1% TritonX-100 and PowerBlock. Primary staining lasted 1 hour, followed by two 15-minute washes with 0.025% Tween 20 in PBS (Sigma, Cat: P1379) and a single 15-minute wash in PBS. Secondary antibody containing Alex Fluor 488, 594, or 647 against the primary antibody was added to a 1:1 solution of 0.1% TritonX-100 and PowerBlock. Secondary antibody staining lasted 45 minutes, followed by two 30-minute washes in 0.025% Tween 20 in PBS (Sigma, Cat: P1379) and a single 30-minute wash in PBS. Stained gel pieces were mounted on a slide with Fluoromount-G mounting solution with DAPI (ThermoFisher Cat:00495952). Fluorescent images were taken with an LSM880 inverted confocal microscope.

The following primary antibodies were used for staining gels: CD45 (Abcam, Cat: ab8216), CD163 (Cat: PA5–78961), and COL1 (Cell Signaling, Cat: 72026S).

### Statistical analysis

All data are shown as mean ± standard deviation, unless otherwise specified. Statistical testing was performed in GraphPad Prism v9 unless otherwise specified. For two-group comparisons, unpaired t-tests were used. For multi-group comparisons, one-way ANOVAs were used with Bonferroni’s *post hoc* corrections to compare groups; p < 0.05 conferred statistical significance for all tests. * P < 0.05; ** P < 0.01; *** P < 0.001; **** P < 0.0001.

## Supplementary Material

1

## Data Availability

The scRNA-seq dataset generated and analyzed during the current study will be available in the Gene Expression Omnibus (GEO) by the time of publication.
