# Supplementary material for: A tension offloading patch mitigates dermal fibrosis induced by pro-fibrotic skin injections
Source: Res Sq. 2024 Mar 1:rs.3.rs-3915097. Preprint. [Version 1] doi: 10.21203/rs.3.rs-3915097/v1 (PMC10925431; doi:10.21203/rs.3.rs-3915097/v1)
Supplement: 1 [file NIHPPrs3915097V1-supplement-1.pdf]

Supplementary Figures

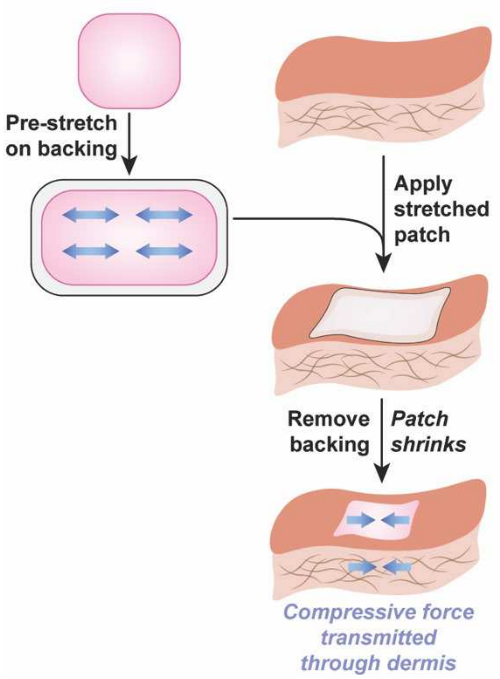

Supplementary Fig. 1: Mechanism of action of tension offloading patch. Schematic depicting mechanism of patch application. The elastomeric silicone patch is pre-stretched via expansion of the attached cardboard backing (left column). The pre-stretched patch is then applied to the skin (right column, top two panels). When the cardboard backing is removed, the patch shrinks back to its original, unstretched size, applying an overall compressive force to the skin (right column, bottom panel).

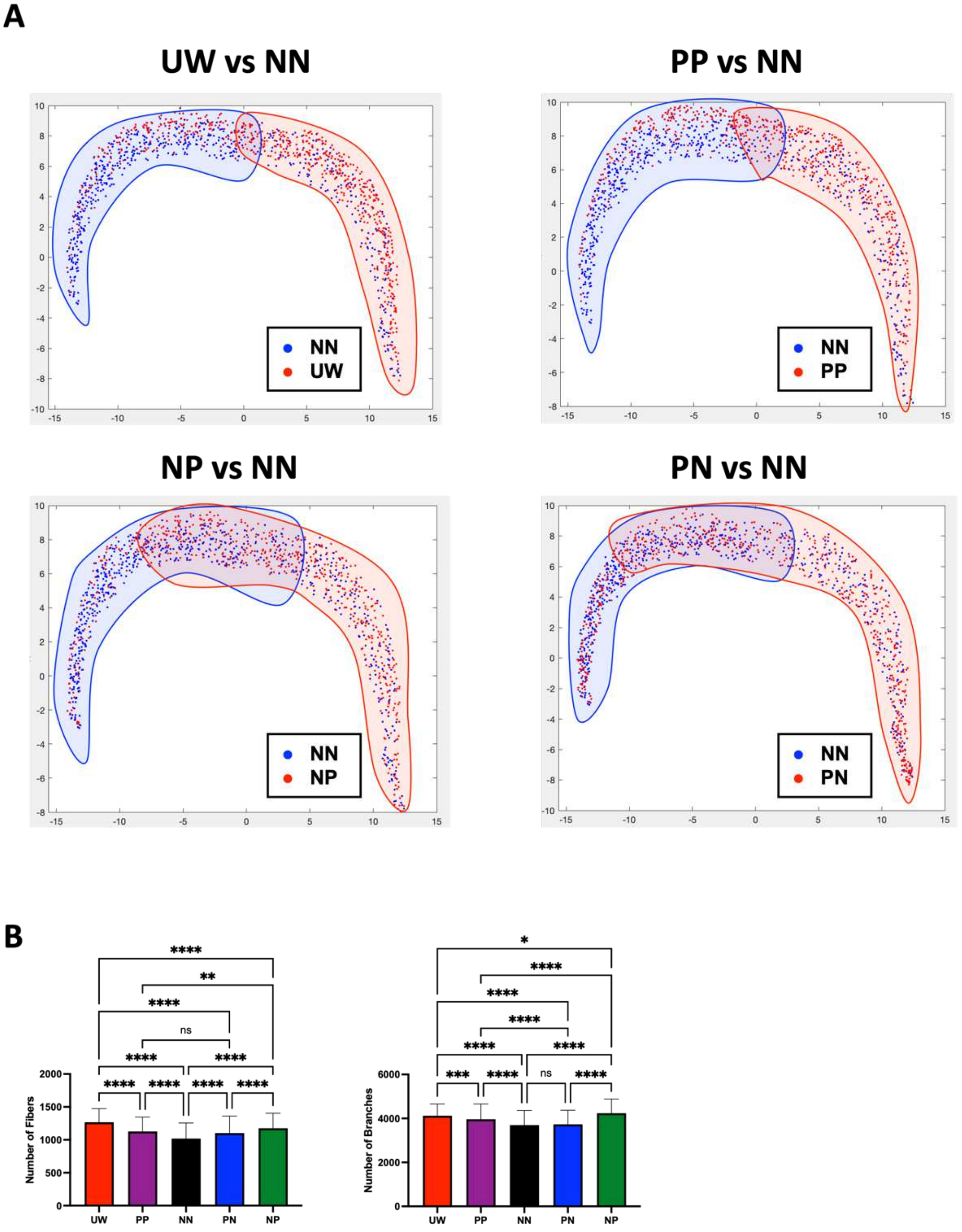

Supplementary Fig. 2: Quantitative extracellular matrix (ECM) analysis of porcine dermal fibrosis model. (A) Uniform manifold approximation and projection (UMAP) displaying unwounded skin (UW) and individual patch-treated experimental groups (PP, NP, and PN) plotted against non-patch-treated, bleomycin-injected skin (NN). Shaded regions highlight distribution of points corresponding to each experimental condition across UMAP space. **(B)** Quantification of

average number of collagen fibers (left) and number of fiber branchpoints (right) per picrosirius red image analyzed for each experimental group as determined by quantitative ECM analysis. (B) Data shown as mean ± standard deviation (SD). *ns*, not significant; *$P < 0.05$; **$P < 0.01$; ***$P < 0.001$; ****$P < 0.0001$. $n = 3$ regions of skin per experimental group from each of 2 pigs unless otherwise specified.

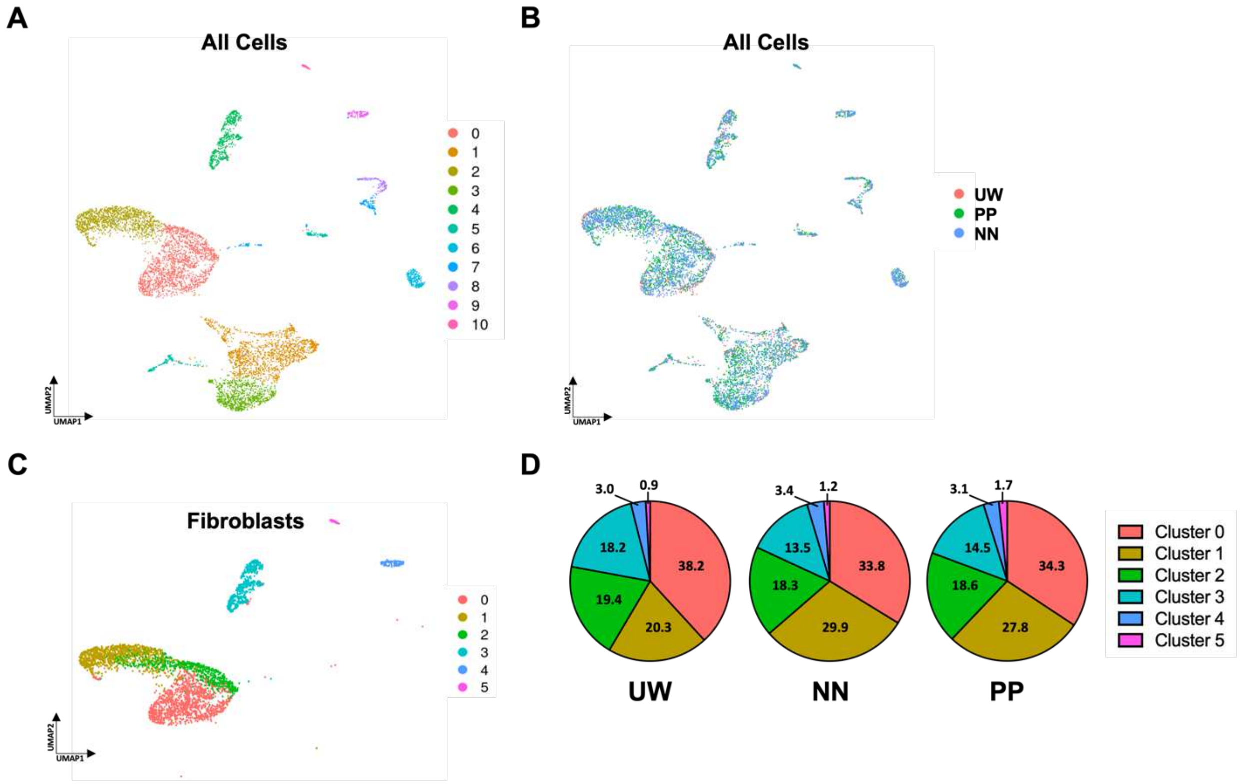

Supplementary Fig. 3: Single-cell RNA-sequencing (scRNA-seq) analysis of skin cells and fibroblast subtype analysis in porcine dermal fibrosis model. (A-B) UMAP of all cells colored by Seurat cluster (A) or experimental group (B). **(C)** UMAP of fibroblasts colored by Seurat cluster (0-5). **(D)** Relative representation of fibroblasts belonging to Seurat clusters 0-5 in UW, PP, and NN dermis.

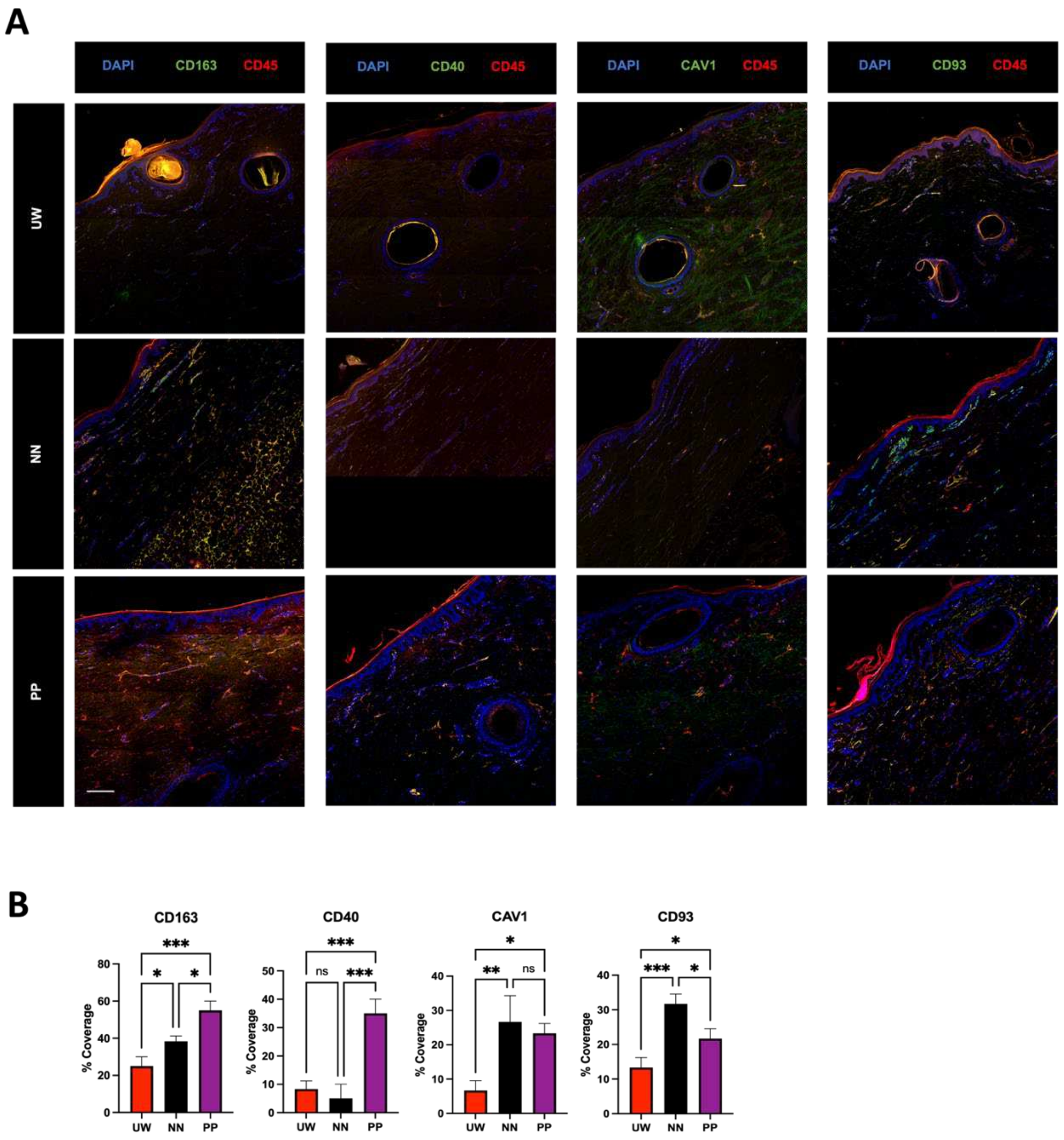

Supplementary Fig. 4: Immunofluorescent (IF) staining analysis of myeloid subtype markers in porcine skin. (A) IF staining of cluster of differentiation (CD) 193, CD40, caveolin 1 (CAV1), and CD93 (all, green signal), co-stained with CD45 (red signal) and DAPI (4′,6-diamidino-2-phenylindole) nuclear counterstain (blue signal), in indicated experimental conditions. Scale bars, 75 µm. **(B)** Quantification of CD163, CD40, CAV1, and CD93 expression from IF staining. Data shown as mean ± standard deviation (SD). *ns*, not significant; *$P < 0.1$; ** $P < 0.01$; ***$P < 0.001$.

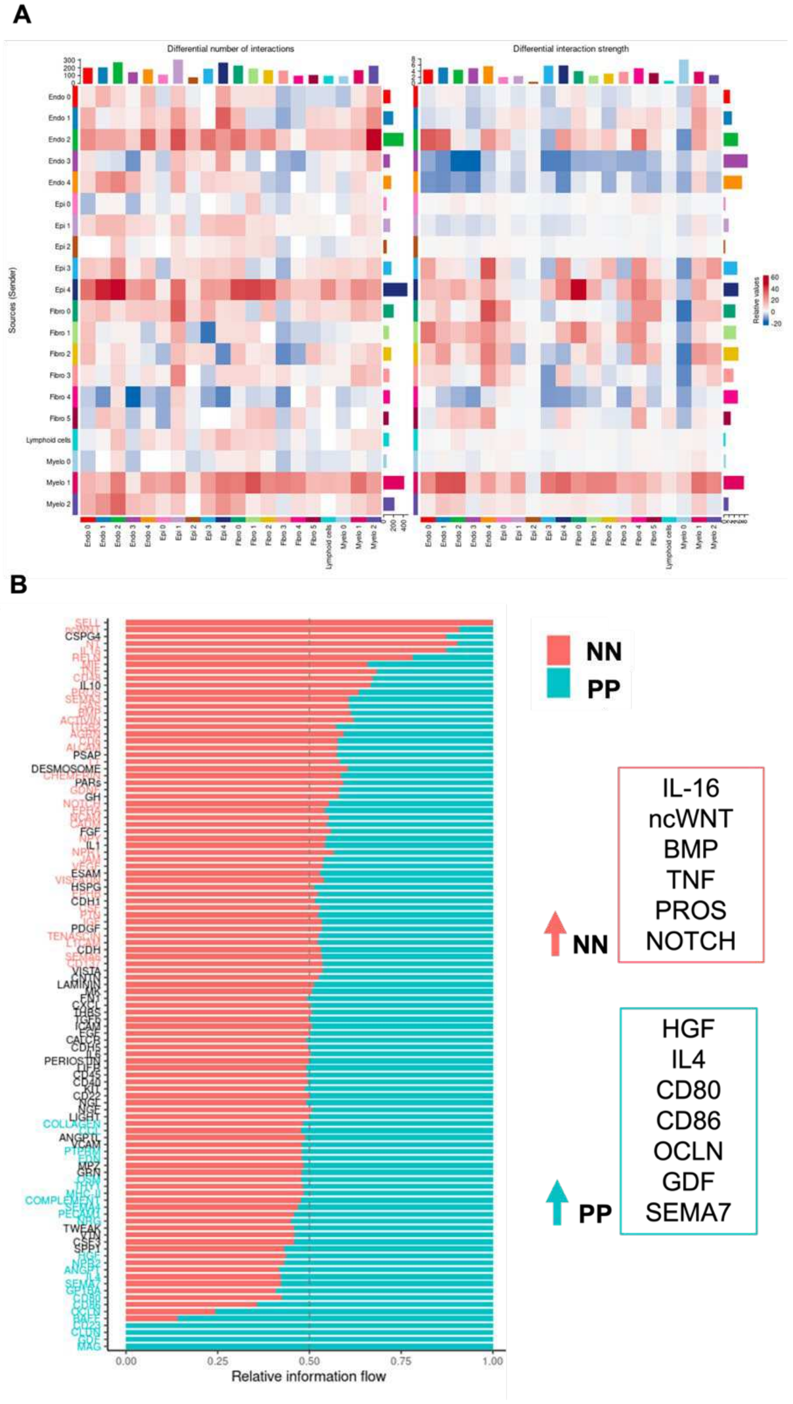

Supplementary Fig. 5: CellChat analysis of intercellular signaling in porcine fibrosis model. (A) Heatmaps showing differential cell-cell interaction numbers (left) and strength (right) in cells from PP skin relative to those from NN skin (blue shades, decreased cell-cell signaling in PP dermis compared to NN dermis; red shades, increased cell-cell signaling in PP dermis compared

to NN dermis). **(B)** Left panel, relative information flow for each significant pathway identified via CellChat. X-axis shows percentage of interactions per pathway identified, comparing NN dermis to PP dermis. Top signaling pathways colored in red are enriched in NN dermis, while those in blue are enriched in PP dermis. Right panels highlight specific pathways enriched in NN dermis (top right panel, red box) while those in the blue box highlight specific pathways enriched in PP dermis (bottom right panel, blue box).

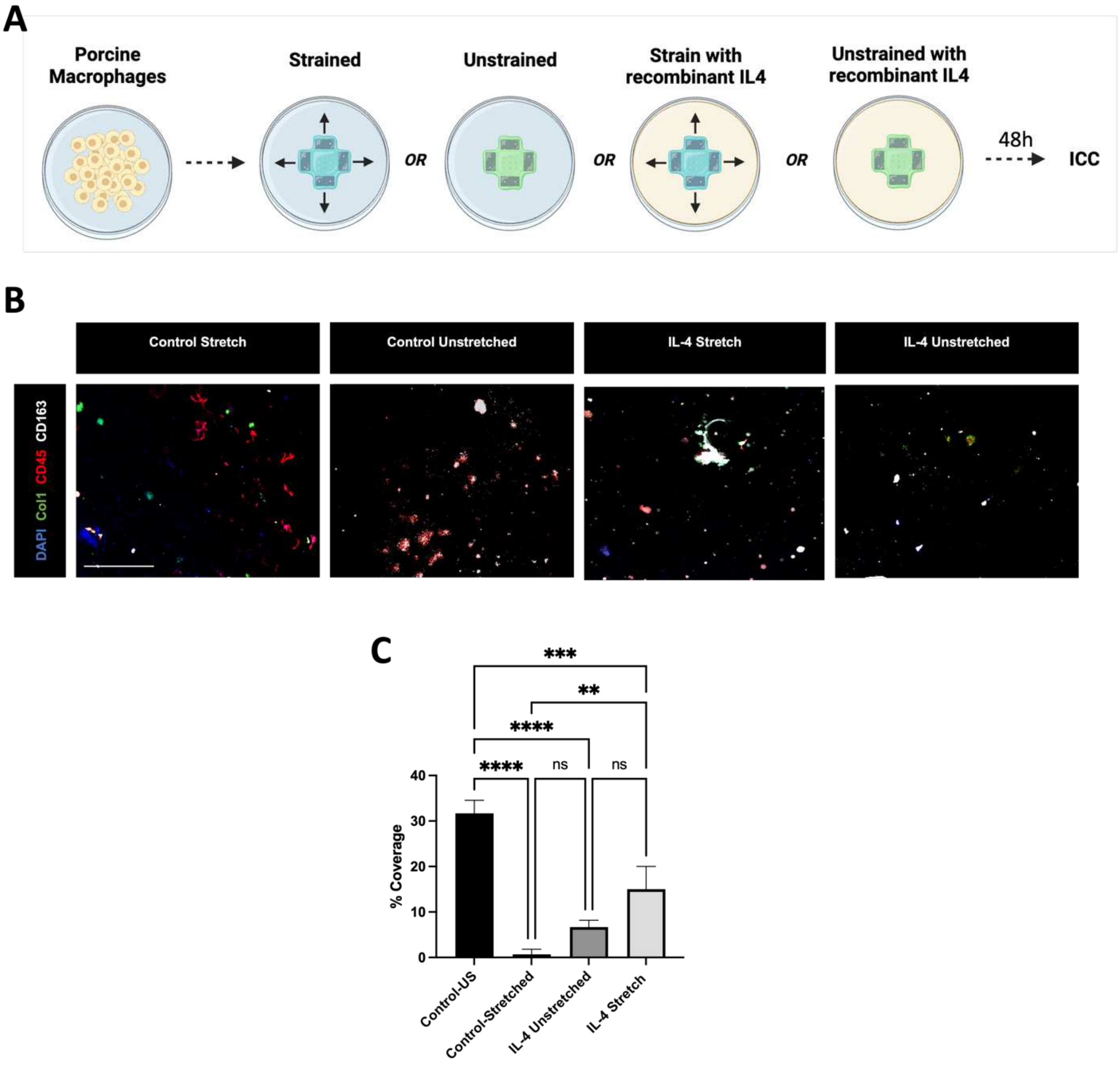

Supplementary Fig. 6: *In vitro* analysis of the effects of mechanical strain and IL4 on porcine macrophage subtype identity. (A) Schematic showing porcine macrophage isolation, culture, and mechanomodulation experiments. **(B)** Immunocytochemistry (ICC) of macrophages isolated from red Duroc pig skin, following 3D hydrogel culture with applied stretch (first column), no stretch (second column), applied stretch with recombinant interleukin 4 (IL4; third column), or no stretch with recombinant IL4 (fourth column) showing representative staining for CD163 (white signal), CD45 (red signal), and collagen type 1 (COL1, green signal). DAPI (4′,6-diamidino-2-

phenylindole), nuclear counterstain (blue signal). Scale bar, 50 μm. **(C)** Quantification of CD163 expression for each experimental condition based on ICC staining. Data are shown as mean ± standard deviation (SD). *ns*, not significant; ** $P < 0.01$; ***$P < 0.001$; ****$P < 0.0001$.

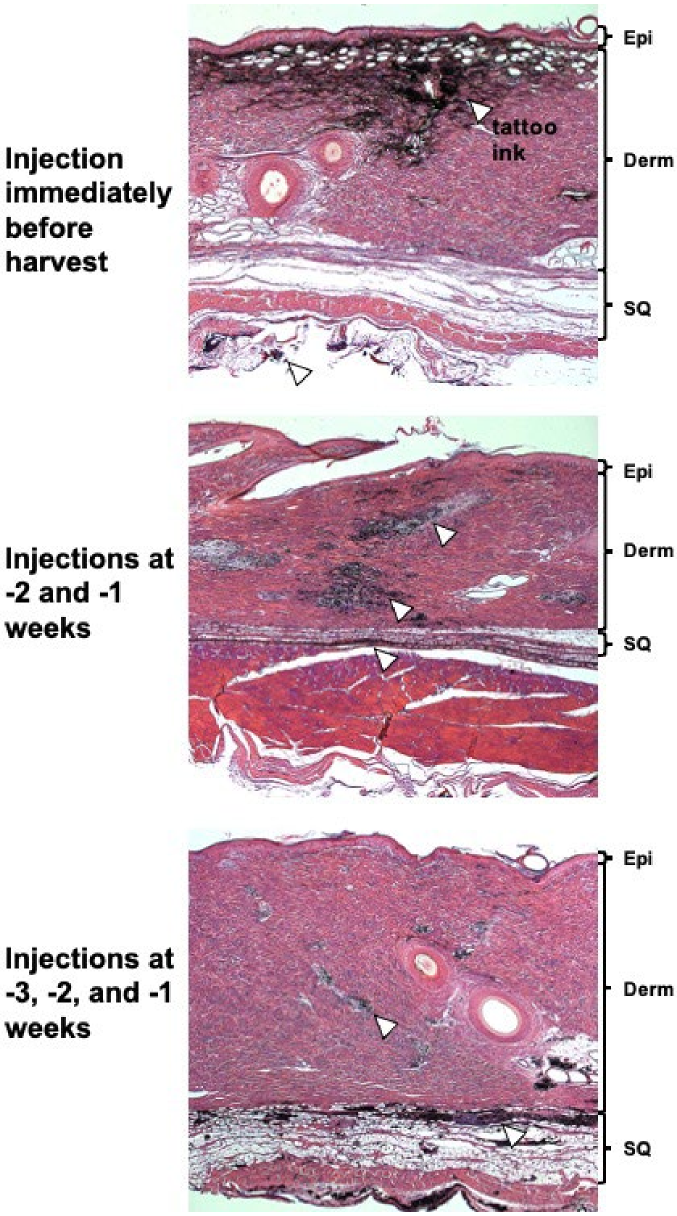

Supplementary Fig. 7: India ink analysis of localization of intradermally injected material. H&E histology following the injection of India ink (750 µl per injection, diluted 1:1 in sterile saline) intradermally either at the time of harvest (top panel), two weeks and one week before harvest (middle panel), or three, two, and one weeks before harvest (bottom panel) to demonstrate intradermal and subcutaneous localization of injected material. White arrows highlight injected India ink tattoo.
